# Microfluidic-based prostate cancer model for investigating the secretion of prostate-specific antigen and microRNAs in vitro

**DOI:** 10.1038/s41598-023-38834-y

**Published:** 2023-07-19

**Authors:** Adventina Padmyastuti, Marina Garcia Sarmiento, Maria Dib, Jens Ehrhardt, Janosch Schoon, Maryna Somova, Martin Burchardt, Cindy Roennau, Pedro Caetano Pinto

**Affiliations:** 1 Department of Urology, University Medicine Greifswald, Fleischmannstraße 8, 17475 Greifswald, Germany; 2 Department of Ear, Nose and Throat Surgery, University Medicine Greifswald, Fleischmannstraße 8, 17475 Greifswald, Germany; 3 Department of Obstetrics and Gynecology, University Medicine Greifswald, Fleischmannstraße 8, 17475 Greifswald, Germany; 4 Center for Orthopaedics, Trauma Surgery and Rehabilitation Medicine, University Medicine Greifswald, Fleichmannstraße 8, 17475 Greifswald, Germany

**Keywords:** Oncology, Urology, Cancer models, Urogenital models

## Abstract

The study of prostate cancer in vitro relies on established cell lines that lack important physiological characteristics, such as proper polarization and expression of relevant biomarkers. Microphysiological systems (MPS) can replicate cancer microenvironments and lead to cellular phenotypic changes that better represent organ physiology in vitro. In this study, we developed an MPS model comprising conventional prostate cancer cells to evaluate their activity under dynamic culture conditions. Androgen-sensitive (LNCaP) and androgen-insensitive (PC3) cells were grown in conventional and 3D cultures, both static and dynamic. Cell morphology, the secretion of prostate-specific antigen, and the expression of key prostate markers and microRNAs were analyzed. LNCaP formed spheroids in 3D and MPS cultures, with morphological changes supported by the upregulation of cytokeratins and adhesion proteins. LNCaP also maintained a constant prostate-specific antigen secretion in MPS. PC3 cells did not develop complex structures in 3D and MPS cultures. PSA expression at the gene level was downregulated in LNCaP-MPS and considerably upregulated in PC3-MPS. MicroRNA expression was altered by the 3D static and dynamic culture, both intra- and extracellularly. MicroRNAs associated with prostate cancer progression were mostly upregulated in LNCaP-MPS. Overall dynamic cell culture substantially altered the morphology and expression of LNCaP cells, arguably augmenting their prostate cancer phenotype. This novel approach demonstrates that microRNA expression in prostate cancer cells is sensitive to external stimuli and that MPS can effectively promote important physiological changes in conventional prostate cancer models.

## Introduction

Prostate cancer (PCa) was the second most diagnosed malignancy and the fifth leading cause of oncological mortality among men in 2020^1^. The prevalence and mortality of PCa correlate worldwide with an increase in age, and estimates place the incidence rate over 65 years at 60%^2^. The growth and survival of PCa cells are initially dependent on androgens, namely testosterone, which in their active form bind to the androgen receptor (AR), and regulate the expression of multiple proteins involved in cell proliferation or apoptosis evasion mechanisms. Androgen deprivation therapy (ADT) is a standard, and widely used, PCa therapy. ADT actively downregulates the synthesis of testosterone and therefore suppresses PCa proliferation. Approximately 10–15% of PCa patients develop castration resistance, which offers a poor clinical prognosis, with a median survival time of 12–23 months^34^. Castration-resistant prostate cancer (CRPC) develops when malignant PCa cells proliferate in the absence of androgens, rendering ADT ineffective. CRPC is characterized by an increase of the prostate-specific antigen (PSA) in three consecutive analyses at least one week apart and a PSA level of > 2 ng/ml or tumor radiographic progression following ADT^5^.

PSA is a kallikrein-related serine protease produced by the epithelial cells of the prostate gland. It serves as a biomarker for screening, diagnosis, and follow-up of PCa. Physiologically, PSA secretion facilitates sperm mobility, and its expression is regulated by AR. This biomarker reflects the proliferation of prostate cells, and elevated PSA levels can indicate the presence of cancer cells. However, despite its advantages, PSA lacks diagnostic specificity and does not indicate disease severity or stage. Benign prostate conditions or injuries can also result in elevated PSA levels^6^. This illustrates the need for more specific PCa biomarkers as diagnostic and prognostic tools.

MicroRNAs (miR) have been identified as potential biomarkers for the early diagnosis of CRPC, with the promise to capture this malignancy before the onset of castration resistance ^7^. Several microRNAs (e.g. miR-4417, miR-3687, miR-205) have been described to have a functional role in CRPC progression^8^. Comprehensive studies, both clinical and pre-clinical, are necessary to further explore the role of microRNAs in the molecular transition between PCa and CRPC and to validate their use as clinical biomarkers for CRPC.

Despite the widespread use and contributions of several cell lines in molecular urology research (e.g. AR de-regulation), there are major limitations in their applications in terms of discovery and validation of clinically relevant biomarkers and as robust platforms for drug testing in a pre-clinical set-up, for instance, due to the high variability of biomarker expression in various PCa and CRPC cells^9,10^. Further, cell lines in conventional culture (2D) fail to replicate the heterogeneity characteristic of the tumor microenvironment, do not sustain the typical polarity of epithelial cells, and have restricted or absent expression of key cellular machinery (e.g. adhesion proteins, membrane carriers, metabolic enzymes)^11^.

Advances in micro-physiological systems (MPS) have enabled the introduction of the so-called organs-on-a-chip, where cells are cultured under dynamic conditions. In MPS cells are exposed to diverse external stimuli (e.g. flow, pressure, mechanical stress, etc.) that promote the differentiation of features absent in 2D^12^. Recent studies have shown that MPS substantially improve the function of epithelial cells. Among the notable features consistently absent in 2D cultures that are recovered in organ-on-a-chip models is the secretion of anionic drugs (kidney), gut microbiome enzymatic activity (intestine), and biliary duct formation (liver)^13^. These innovative in vitro platforms are fast being adopted for toxicological and pharmaceutical studies that require a better representation of human physiology ^14^. Recent attempts to demonstrate the effects of dynamic culture on the behavior of prostate cells have yielded promising results. Prostate cells can maintain their viabililty under fluidic conditions^15,16^. A comphrensive study using androgen sensitive LNCaP cells, showed that spheroids in a MPS enhanced cell growth, improved structural integrity, reduced necrotic core formation, and downregulated expression of cell stress genes. Flow-cultured spheroids displayed increased sensitivity to chemotherapy and exhibited a greater transcriptional response^17^.

In this study, we aimed to develop a prostate cancers-MPS (PCa-MPS) model that recreates the epithelial nature of PCa and CRPC cells as well as their PSA and miRNA secretion in-vitro. Commonly used LNCaP cells were used as a model for androgen-sensitive PCa cells and PC3 cells were used as a model for androgen-insensitive PCa cells. Exploring the potential of these cells in an MPS model provides a new and more physiologically accurate tool for the study of PCa biomarkers for potential use in clinical practice and drug development applications.

## Results

### Three-dimensional and MPS conditions alter the morphology of prostate cancer cells

**Figure 1:**
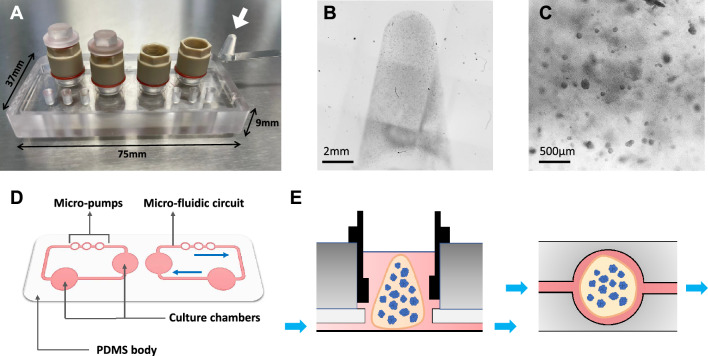
HUMIMIC chip 2 microphysiological system. (**A**) HUMIMIC chip 2 with the chambers of the right perfusion circuit open. An agar/collagen I hydrogel is shown next to the chip (highlighted circle). (**B**) Transmitted light image (2x) of the hydrogel used, where the conical shape of the gel is evident, as well as the embedded cellular 3D structure. (**C**) Detail of a hydrogel (10x) showing the density of LNCaP spheroids. (**D**) Representation of the microfluidic circuits of the HUMIMIC chip 2, which comprises 2 independent circulations with two culture chambers each, encased in a Polydimethylsiloxane (PDMS) body. The circuits are located at the bottom of the chip, with a glass body on top, evident in panel A. (**E**) Representation of a cross-section of a culture chamber with the hydrogel inside. The perfusion is driven from the bottom of the chamber (blue arrows), and flow disperses through the gel and supernatant in cycles. **E:** Representation of the bottom of the chip, which is comprised of a clear microscopy glass that enables direct visualization of the chambers.

**Figure 2:**
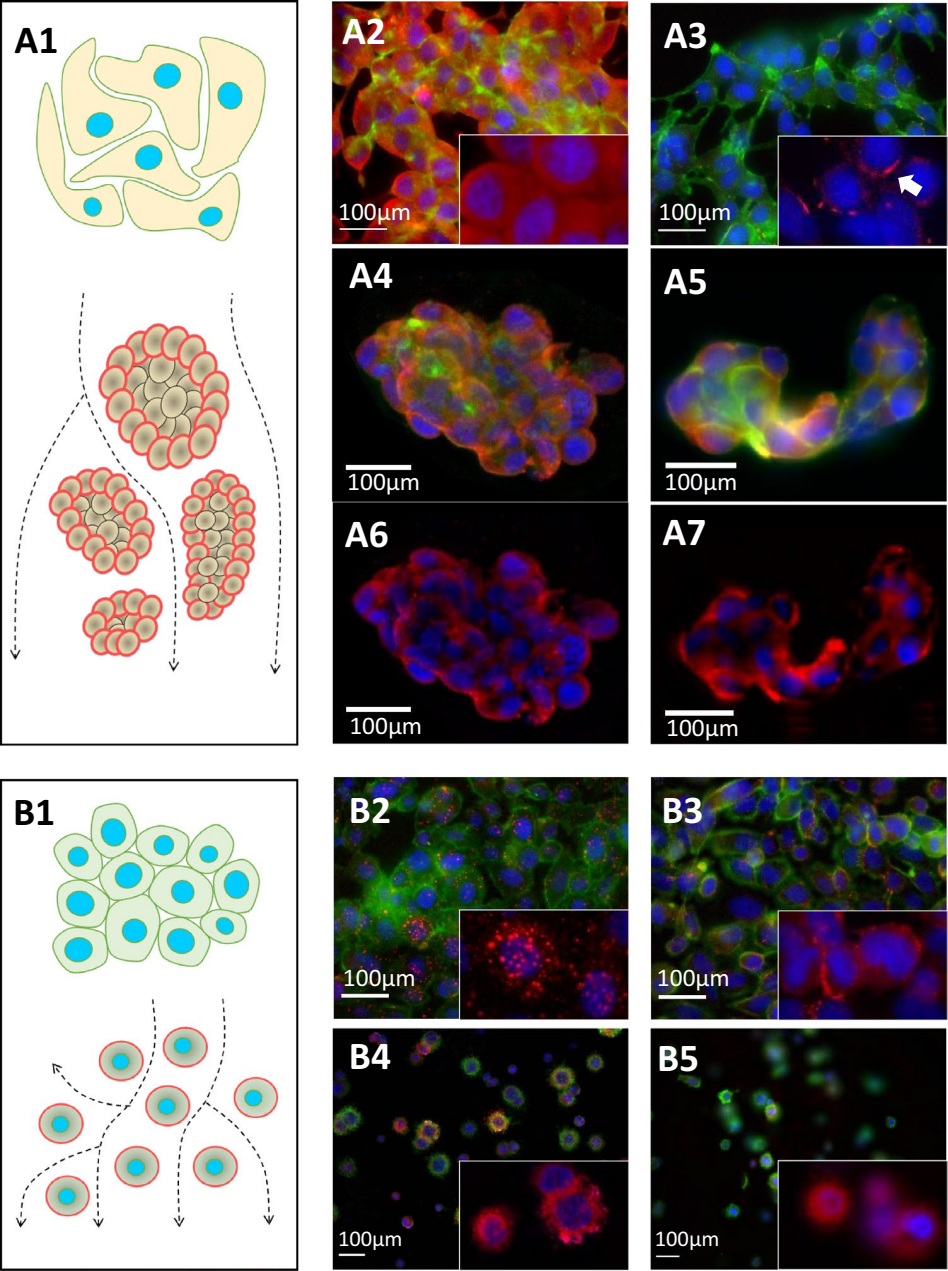
Immunofluorescent characterization of LNCaP and PC3 cells. (**A1**) Schematic representation of the morphology of LNCaP cell in 2D (top) and dynamic culture (bottom). In all images Hoechst33342 (nuclei, blue)) and Phalloidin-488 (f-actin, green) were used as co-stain. The markers of interest were detected using Alexa-555 antibodies (red) (**A2**) Epidermal growth factor receptor (EGFR) is seemingly dispersed in the cytoplasm of LNCaP cells in 2D. (**A3**) Zonula ocludens-1 (ZO1) is present in the cellular boundaries of LNCaP, although to a lesser extent. (**A4**) LNCaP cells in MPS develop into non-hollow spheroids with EGFR predominantly expressed in the outer boundaries of the structures. (**A5**) In MPS**,** the expression of ZO1 in LNCaP cells is pronounced and evident between cells. F-actin filaments also present a more consistent organization in MPS and are predominantly concentrated in the periphery of the cells. (**A6**) EGFR and nuclear stain alone, in LNCaP-MPS. (**A7**) ZO1 and nuclear stain alone, in LNCaP-MPS. (**B1**) Schematic representation of the morphology of PC3 cells in 2D (top) and dynamic culture (bottom). (**B2**) EGFR is concentrated in endosomes dispersed in the cytoplasm of PC3 cells in 2D. (**B3**) ZO1 expression is pronounced in the cellular boundaries of PC3 cells. (**B4**) In MPS, PC3 do not develop into 3D structures and remain as individual cells, suspended in the gel. Nonetheless, EGFR and f-actin are predominately found in the periphery of the cells. (**B5**) ZO1 is dispersed in the cytoplasm of PC3 in MPS, lacking proper membrane localization.

The MPS chip used in our study enables pulsating flow between two culture chambers in a close circuit (Fig. 1D), these stimuli mean that culture media is constantly re-perfused over the cells and that the ECM hydrogel contracts in each perfusion cycle. In both 3D (static) and MPS cultures, LNCaP cells grow into non-hollow spheroids with an apparently increased polarization relative to conventional culture (Fig. 2A1). In LNCaP spheroids, the Epidermal growth factor receptor (EGFR) is expressed predominately on the surface of the cells, in contrast with 2D cultures, where EGFR is dispersed in the cytoplasm (Fig. 2A2, A4-6). The density of the adhesion protein ZO1 is also more substantially pronounced in MPS cultured cells than in 2D, emphasizing the enhanced cohesion of the spheroids (Fig. 2A3, A5-7). PC3 cells did not develop into 3D structures in MPS culture (Fig. 2B1). Nonetheless, EGFR expression is seemingly concentrated in the periphery of the individual cells in the gel (Fig. 2B4). This indicates that the cells augment their polarity relative to 2D cells, where EGFR is dispersed in the cytoplasm (Fig. 2B2). In 2D, PC3 cells adhere to each other, evident from the ZO1 expression in the cell’s boundaries (Fig. 2B3), a feature that is precluded in MPS culture (Fig. 2B4).

**Figure 3:**
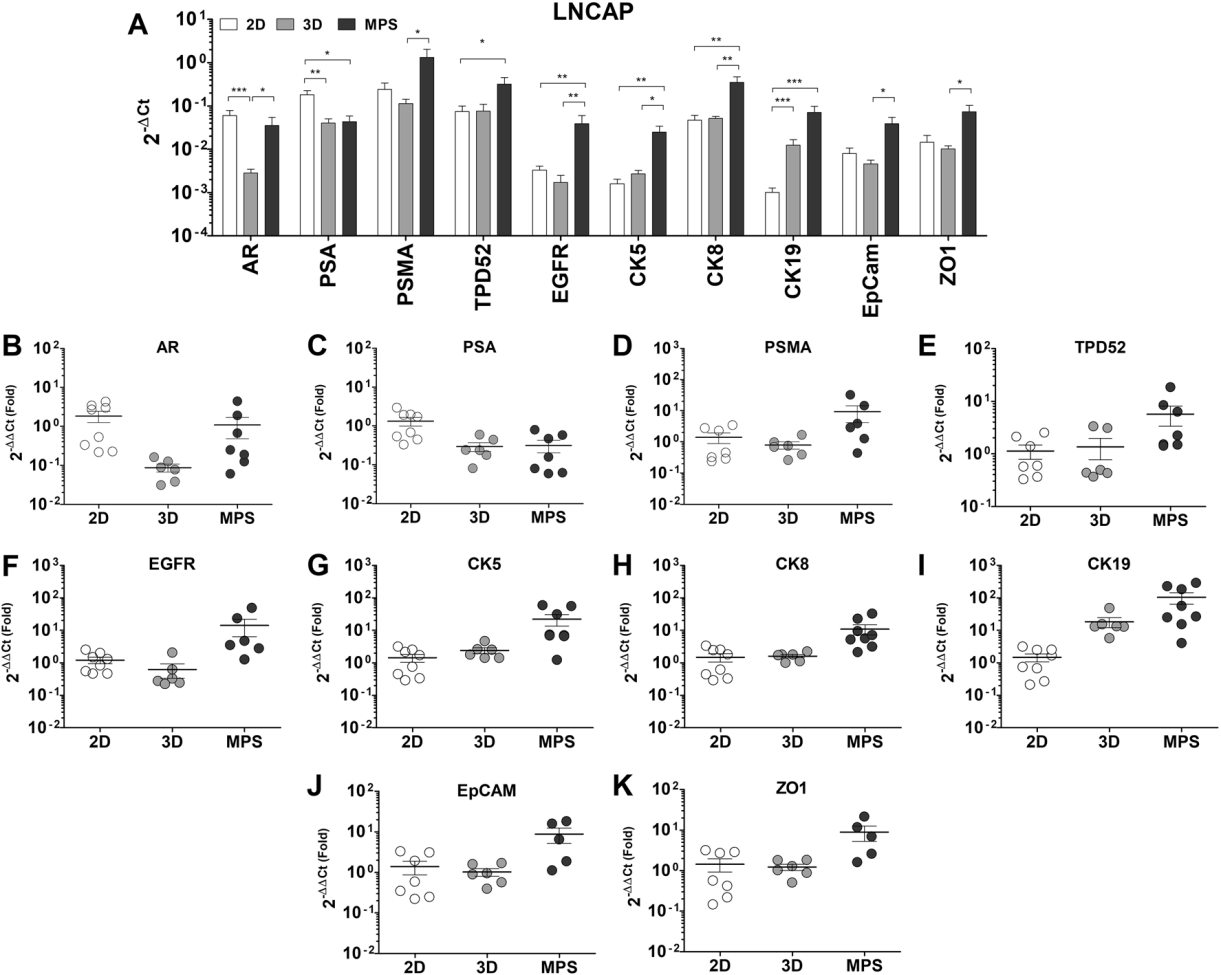
Differences in gene expression between LNCaP cells in 2D, 3D, and MPS: (**A**) Absolute expression values (2^-ΔCt^), presented in a log_10_ scale. There is evident deregulation of several key prostate cancer markers when cells are cultured under 3D -static or -dynamic conditions, relative to conventional culture. Significant statistical differences were found in the expression of all genes analyzed across the three culture conditions tested. This data represents a minimum of six independent samples analyzed using three technical replicates. Each data point represents the average value of the technical replicates. Statistical significances were determined using a two-tailed unpaired t-test (*p < 0.05; **p < 0.01; ***p < 0.001).

### Differential gene expression between conventional, 3D, and MPS cultures

The expression of 10 marker genes relevant to the PCa phenotype was analyzed to evaluate differences between conventional cell culture—microplates -, 3D static cultures, and MPS dynamic cultures. Changes in the expression of these PCa markers underline the impact of the MPS conditions. In LNCaP cells (Fig. 3) all markers analyzed alerted their expression across 3D -static and -dynamic conditions. Notably, the expressions of CK5(27.0 ± 10.7-fold), CK8(12.2 ± 5.0-fold), and CK19(51.9 ± 27.8-fold) are upregulated (Fig. 3G–I), as well as the levels of Epithelial cell adhesion molecule (EpCam; 7.4 ± 3.1-fold) and Zonula occludens-1 (ZO1; 7.5 ± 3.2-fold), in MPS relative to cell culture in 2D (Fig. 3J, K). The upregulation of cytokeratins, components of the cytoskeleton that enable cells to withstand mechanical stresses, reiterates the effects of the dynamic culture in restructuring the LNCaP phenotype. Enhanced gene expression of adhesion proteins is also consistent with the enhanced expression of ZO1 observed in LNCaP spheroids. The expression of AR was only deregulated in 3D culture (Fig. 3B), however, there is a downregulation in the levels of PSA, of about 0.4-fold in both 3D -static and MPS conditions (Fig. 3C). EGFR expression was also upregulated by 19.9 ± 10.9-fold as well as Tumor protein D52 expression (TPD52; 6.2 ± 3.3-fold), a protein reported to mediate the proliferation of PCa^18–20^, but only in MPS conditions (Fig. 3E, F).

**Figure 4:**
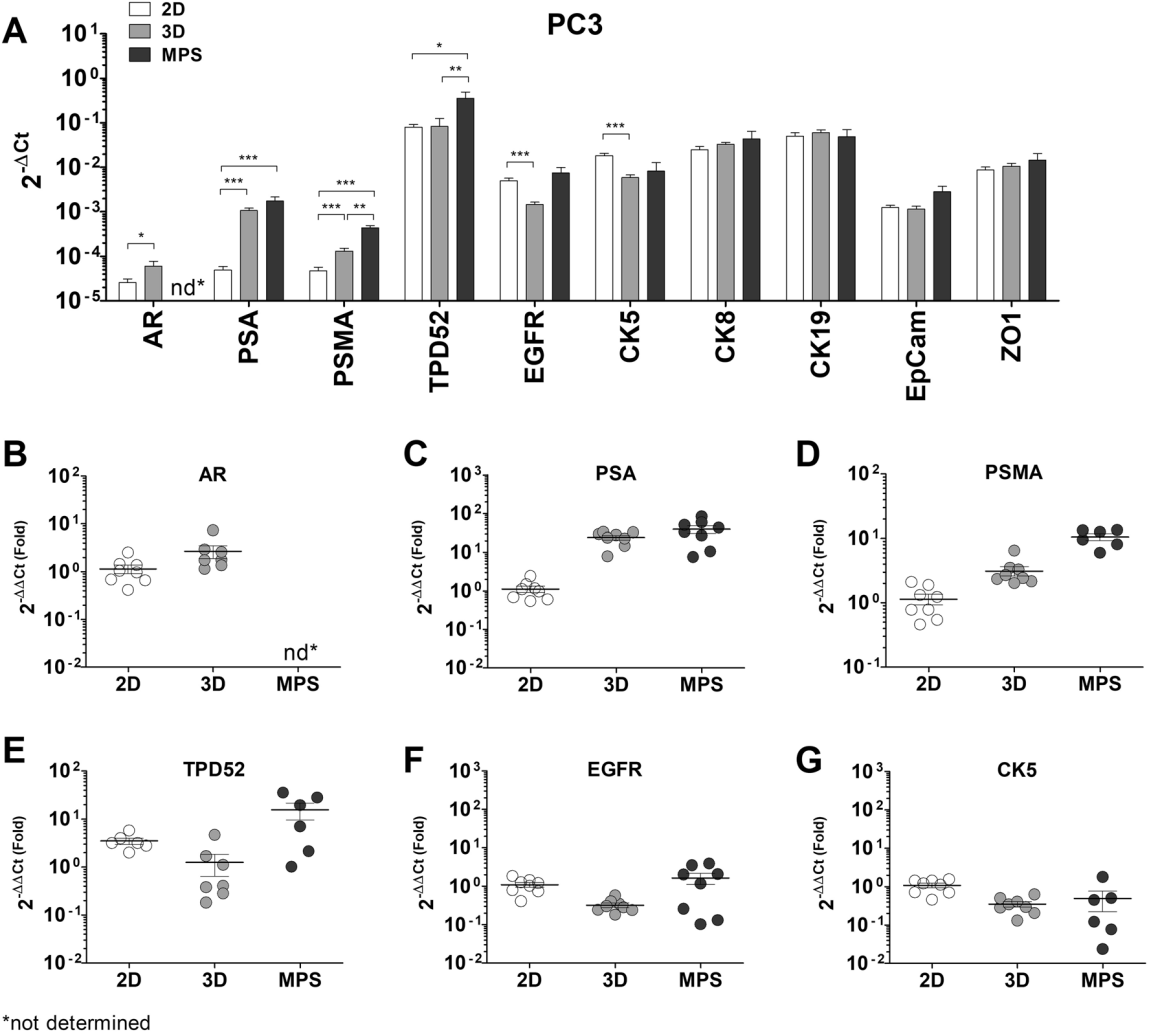
Differences in gene expression between PC3 cells in 2D, 3D, and MPS: (**A**) Absolute expression values (2^-ΔCt^), presented in a log_10_ scale. There is evident deregulation of certain key prostate cancer markers when cells are cultured under 3D -static or -dynamic conditions, relative to conventional culture. Significant statistical differences were found in the expression of AR (**B**), PSA (**C**), prostate-specific membrane antigen (PSMA) (**D**), TPD52 (**E**), EGFR (**F**)**,** and CK5 (**G**). 3D culture under static seems to exert the most differences in gene expression of PC3 cells. This data represents a minimum of six independent samples analyzed using three technical replicates. Each data point represents the average value of the technical replicates. Statistical significances were determined using a two-tailed unpaired t-test (*p < 0.05; **p < 0.01; ***p < 0.001).

The effects of 3D -static and -dynamic culture in the gene expression of PC3, also reveal crucial changes (Fig. 4). The limited changes in the levels of cytokeratins and adhesion molecules are consistent with the lack of differentiation of PC3 cells in 3D. Interestingly, the expression of TPD52 (Fig. 4E) was only upregulated in MPS culture, to 15.1 ± 5.9-fold, and AR expression is slightly upregulated in 3D-static conditions (1.9 ± 0.6-fold) but absent altogether in MPS (Fig. 4B). The expression of PSA is substantially upregulated in both 3D-static and MPS (Fig. 4C), to similar levels (about 30-fold). PSMA levels saw an enhanced upregulation under 3D conditions (Fig. 4D), with the highest increase observed in MPS cultures (10.9 ± 1.4-fold).

**Figure 5:**
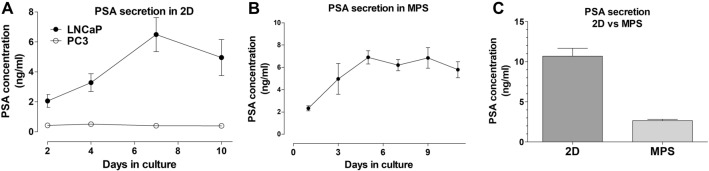
PSA secretion. (**A**) In 2D conventional culture PSA levels in PC3 are marginal and unaltered over time, in culture. In contrast, PSA secretion in LNCaP cells is seen increasing over the culture period. Cells were culture in a 24-well format with 500µL of culture media (**B**) Secretion of PSA over 11 days in LNCaP-MPS, with samples collected in 2–3 days intervals. PSA levels increase up to day 5 in culture and are maintained constant afterward. Cells were culture at a density of 100 000 cells per hydrogel in a volume of 250µL. (**C**) PSA levels in LNCaP-MPS, after 4 days in culture, are substantially lower than the levels determined for 3D static and 2D culture when the cells were cultured for the same period, cell density and media volume.

### PSA secretion in MPS

LNCaP cells, in MPS, can sustain the expression of PSA over 10 days in culture (Fig. 5). PSA levels slightly increased until day 5 in culture and remained constant the following 6 days. The initial increase is likely due to the proliferation of the cells in the hydrogel, considering that the cells form aggregates from a single-cell suspension. The elevation in PSA levels corresponds to cell proliferation during aggregate formation, as PSA secretion is widely acknowledged to be closely associated with prostate cell growth. PSA levels in MPS are lower than the levels detected in the supernatant of LNCaP, culture in conventional 24-well plates using the same cell density.

### microRNA expression and secretion in MPS

The expression of 4 microRNA reported to be extensively deregulated in PCa was analyzed. In clinical samples, both the expression of miR-3687 and miR-4417 is seen upregulated in CRPC tissue, while miR-205 is consistently downregulated^21^. Mir-26a was selected considering its presence in PCa tissues, but so far unclear biological effects. Additionally, a protemics based analysis of cellular processes regulated by miR-3687 and miR-4417, indicates that these miR are associate with extracellular vesicles, which may impact its secretion by PCa cells^22^.

**Figure 6:**
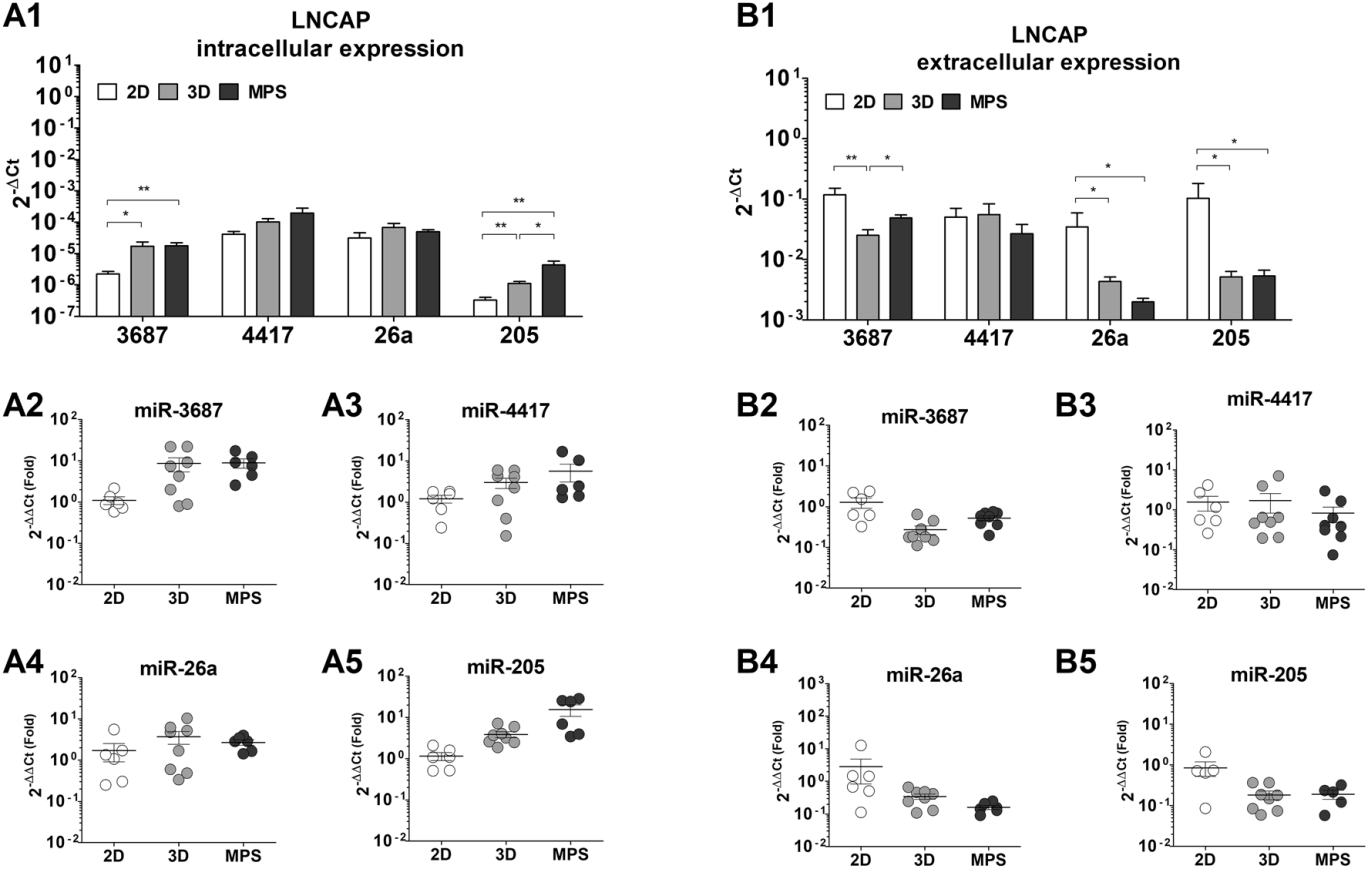
MicroRNA expression in LNCaP cells. (**A1**) Absolute expression values (2^-ΔCt^), presented in a log_10_ scale for microRNA levels determined in cell samples (intracellular). There is evident deregulation of prostate cancer-associated microRNAs when LNCaP cells are cultured under 3D -static or -dynamic conditions, relative to conventional culture. The levels of miR-3667 (**A2**), miR-4417, and miR-205 (**A5**) are seemingly upregulated while the expression of miR-26a is unaltered (**A4**)**.** In supernatant samples (**B1**)**,** only the levels of miR-3687 are seemingly deregulated (**B2**)**.** The expressions of miR-4417 (**B3**), miR-26a (**B4**), and miR-205 (**B5**) were determined to be considerably variable in the LNCaP supernatant. This data represents a minimum of six independent samples analyzed using three technical replicates. Each data point represents the average value of the technical replicates. Statistical significances were determined using a two-tailed unpaired t-test   (*p < 0.05; ***p < 0.001).

Their expression was also found to be impacted by the introduction of the 3D static culture and MPS dynamic culture when compared to 2D culture conditions. In LNCaP (Fig. 6), the intracellular expression of miR-3687 is upregulated to similar levels (about ninefold) in both 3D static and MPS conditions (Fig. 6A2). MiR-205 is seemingly the most upregulated miR, with its levels enhanced by 15.3 ± 4.8-fold in MPS, in comparison to cells cultured in 2D (Fig. 6A5). MicroRNAs could also be detected in the LNCaP supernatant. The extracellular levels of miR-3687 are reduced by approximately 0.5-fold on both 3D static and MPS conditions (Fig. 6B2). miR-205 saw the most deregulation in extracellular levels, relative to 2D, with 0.16 ± 0.05-fold in 3D static and 0.3 ± 0.16-fold in MPS conditions (Fig. 6B5) .

**Figure 7:**
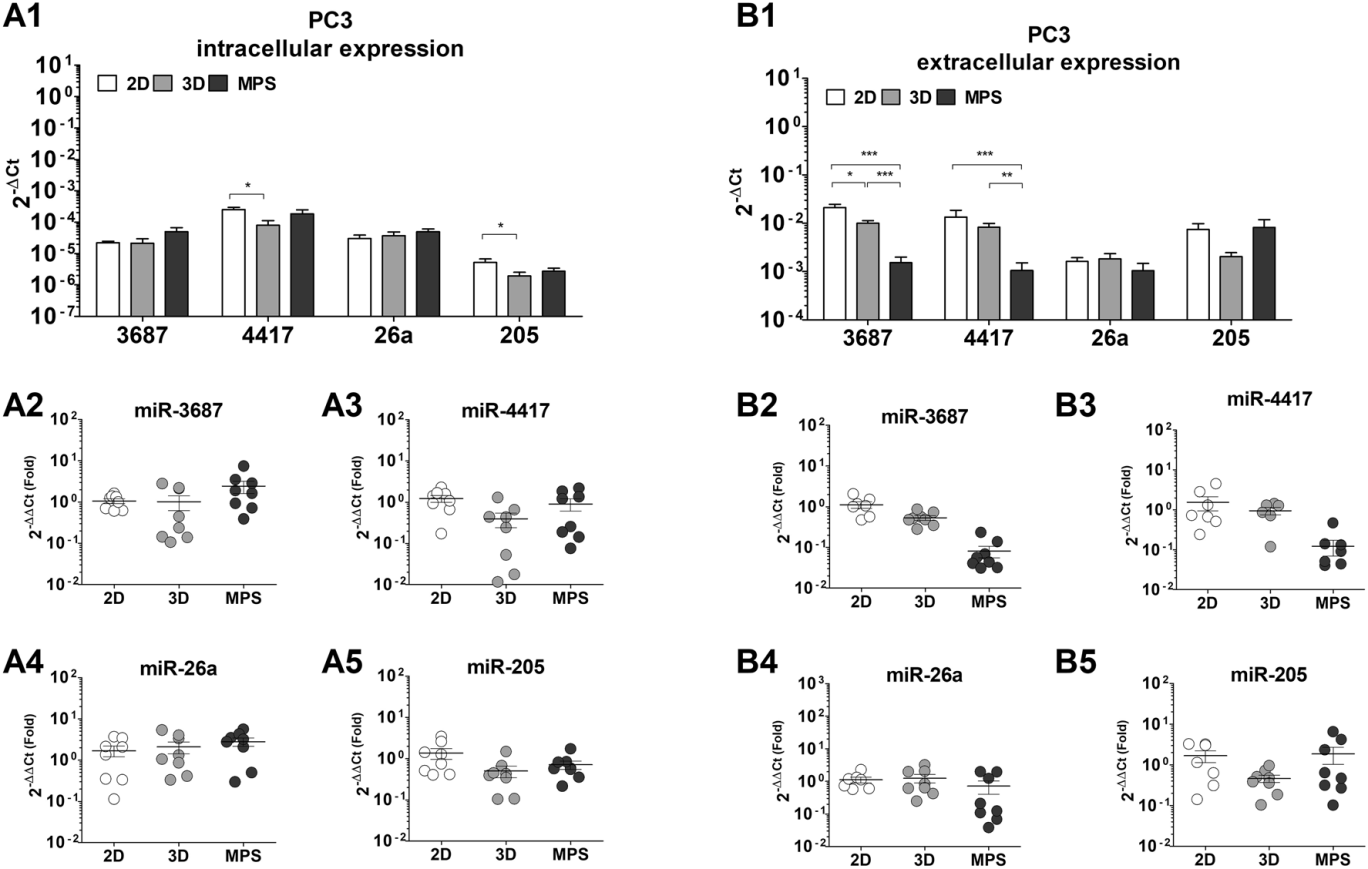
MicroRNA expression in PC3 cells. (**A1**) Absolute expression values (2^-ΔCt^), presented in a log_10_ scale for microRNA levels determined in cell samples (intracellular). Evident deregulation of intracellular prostate cancer-associated microRNAs—miR-3687 (**A2**), miR-4417 (**A3**)**,** and miR-205 (**A5**) was observed in PC3 cells after 3D -static or -dynamic culture condition. The expressions of miR-26a (**B4**) and miR-205 (**B5**) were determined to be considerably variable in PC3 supernatant samples. This data represents a minimum of six independent samples analyzed using three technical replicates. Each data point represents the average value of the technical replicates. Statistical significances were determined using a two-tailed unpaired t-test (*p < 0.05; ***p < 0.001).

In PC3 cells (Fig. 7), the intracellular expression of miR-3687 in MPS is seemingly upregulated by a 2.4 ± 1.0-fold relative to 2D (Fig. 7A2). miR-4417 is seemingly only deregulated under 3D static conditions, by 0.3 ± 0.2-fold (Fig. 7A3). miR-205 is deregulated by approximately 0.5-fold in both 3D and MPS conditions (Fig. 7A5). In terms of extracellular expression, miR-3687 was substantially deregulated in both 3D static and MPS conditions, to 0.5 ± 0.2-fold and 0.08 ± 0.06-fold, respectively (Fig. 7B2). miR-4417 is seem only deregulated in MPS, to 0.1-fold, relative to 2D culture (Fig. 7B3). Due to the fluctuations of miR-26a and miR-205 levels in both intracellular and extracellular conditions of PC3, it is not possible to establish any distinctions between various culture formats (Fig. 7B4, B5).

## Discussion

In recent years, the introduction of MPS models has demonstrated that increasing the complexity of the culture environment, by enabling perfusion and other external stimuli, such as contraction, can significantly change the phenotype of cells. MPS models recreating the functional units of several organs^23–25^ (e.g. kidney, liver, lung, intestine) have now been validated and the number of applications for biomedical and drug development purposes is fast expanding^26,27^. Cancer models have also gathered substantial attention, in particular, because MPS can potentially replicate the microenvironment of tumors and offer a tool to study cancer progression^28,29^ (e.g. metastasis formation, vascularization) and test new classes of drugs in far more human-relevant systems when compared to conventional models. Despite advances in the characterization of cancer-MPS for biomedical applications, the development of PCa and CRPC models based on MPS and their potential impact on the pre-clinical research of these urological cancers is still limited^16,30^. In this study, we show that widely used PCa and CRPC representative cell lines respond to flow and mechanical stress and alter their morphology, gene expression, and secretory profile in a dynamic culture, using the HUMIMIC chip2 which enables re-circulating flow between two culture chambers.

The hydrogels used enable flow through the matrix, cell proliferation and to withstand the cyclic perfusion without compromising their integrity. Concurrently, these hydrogel were also optimized to facilitate the homogeneous and consistent culture of PCa cells, and minimize variability derived from differences in cluster size and cell proliferation over the culture period. These properties ensured that cells were not released from the gel to the supernatant and that secretions (i.e. PSA, microRNA) could be released extracellularly. This was achieved by using a mixture of agar and collagen I, where the agar provides structural integrity and the collagen a substrate for cell adhesion. Although the use of collagen I may influence the proliferation of PCa cells^31^, it comprises ¼ of the total volume of the hydrogel. Moreover, the use of low-density collagen I (3–4 mg/ml) also contributes to limiting its biological effects on the cells. To minimize any potential wide-range data variability, a commonly reported issue in MPS based assays^27,32^, a minimum of six independent experiments were performed for all conditions. In addition, individual samples were analyzed using three technical replicates.

Under these conditions, LNCaP cells developed into relatively round-shaped spheroids, and arguably the spheroid size was limited by the density of the hydrogel (Fig. 1B,C). These morphological changes were supported by the upregulation of the cytoskeleton at the gene level in both 3D static and MPS conditions. In epithelial cells, cytokeratins strengthen the intermediate filaments of the cytoskeleton. Our findings show that MPS conditions exert a more pronounced upregulation of cytokeratins, relative to 3D static cultures. Adhesion proteins, which maintain the coherence of multi-cellular structures only seem upregulated in MPS, alongside EGFR and TPD52. Incidentally, the upregulation of cytokeratins^33^, EpCam^34^, EGFR^35^, and TPD52^18^ is associated with PCa progression, both clinically and in vitro. In the MPS model, it could indicate that the LNCaP cells exhibited an augmented PCa phenotype. Most interestingly is the downregulation of PSA in both 3D static and MPS conditions, and the fact that only in MPS is PSA secretion reduced, relative to 2D culture. These findings indicate that dynamic culture, rather than a 3D environment in itself, precludes PSA secretion. The inhibition of PCa cell growth was shown to be correlated with reduced PSA mRNA levels^36^, which could be related to a limitation in the growth of LNCaP cells, encased in the hydrogel in MPS. Nonetheless, the causes behind the overall downregulation of PSA in the MPS relative to 2D and 3D cultures remains unclear. While PSA levels in 3D static culture are comparable to those in 2D culture, it can be speculated that this decline is related to the introduction of fluid flow in the culture. However, this downregulation at the gene level does not correspond to AR expression, indicating the absence of a direct correlation. In our study, we focused on capturing extracellular PSA secretion and gene expression, but it is important to consider analyzing intracellular levels of PSA in future studies to gain further insights into its expression. In contrast, although PSA is not secreted by PC3 cells under all culture conditions, its expression is upregulated in 3D static and MPS cultures. Notably, under fluidic conditions, the expression of PSA is decreased in androgen-sensitive cells (LNCaP), while increased in androgen-insensitive cells (PC3). Furthermore, our analysis revealed that fluidic conditions enhance cytoskeletal and adhesion elements in LNCaP cells but not in PC3 cells. This indirect observation suggests a potential connection between the reorganization of cellular architecture and PSA expression.

On the other hand, in PC3 cytoskeleton elements, adhesion proteins, and EGFR were not upregulated, which reflects the fact that these cells did not develop into 3D structures. The hydrogels used in this study were optimized using LNCaP cells. These cells were selected to assess and optimize the 3D cell culture due to their tendency to develop aggregates within gels^37^. Since the matrix was not specifically optimized for the 3D culture of PC3 cells, it is probable that the choice of hydrogels has contributed to the absence of PC3 3D structures. PSA secretion was not detected in PC3 cells in both 2D, 3D static, and MPS culture, however, in 3D and MPS conditions its mRNA level is substantially upregulated, along with the expression of PSMA. These findings show a 3D environment alone already impacts the expression of these prostate-specific markers. However, the physiological significance of this observation remains elusive. TPD52 expression is upregulated in MPS but not 3D culture, this fact may be associated with the observed upregulation of PSA and PSMA. Androgens have been reported as positive regulators of TPD52^38^, similar to PSA and PSMA. However, in PC3 cells, androgen receptor (AR) activity is androgen-independent. The introduction of a 3D environment and fluidic culture is likely to enhance AR transcriptional activity, leading to the upregulation of TPD52, PSA, and PSMA. This increase in activity should result in PC3 proliferation, which was not observed. As previously mentioned, the hydrogel properties may be responsible for this lack of proliferation.

In terms of microRNA expression, differences were also evident between 2D, 3D static, and MPS conditions. The intracellular expressions of miR-3687 and miR-205 in LNCap cells is upregulated in 3D cultures relative to 2D. These findings mimick the observations found in clinical samples, where an enhanced expression of both these miR was associated with CRPC progression^39–41^. In particular, miR-205 plays a role in the downregulation of AR activity, which could be implicated in the reduced PSA levels observed in MPS. The reduced expression of MiR-205 correlates with the occurrence of metastases in CRPC patients. It specifically binds to the untranslated region of the AR gene, resulting in a reduction in both transcript and protein levels^41^. Any potential effects of the 3D and MPs conditions in miR-4417 and miR-26a levels were skewed by the variability in our results. On the other hand, the extracellular levels of miR-3697, miR-26a, and miR-205 are downregulated in 3D conditions, relative to 2D. These findings indicate that both 3D static and MPS cultures have comparable effects on altering the secretion profiles of PCa. Therefore, transitioning from a 2D to a 3D environment appears to have the most significant influence on miR expression, in LNCaP cells. In PC3 there is high variability in the intra- and extra-cellular expression of the miR analyzed, which could be an indication that their expression is unstable or inherently heterogeneous. The stability of miR depends on a variety of reasons, believed to be related to their nucleotide sequence, which influences structural characteristics and binding to adaptor proteins, and compartmentalization in the cells (e.g. endosomes, extracellular vesicles)^42^. A better understanding of these factors could benefit a proper extrapolation of the significance of their secretion. The only clear effect is the deregulation of miR-3687 and miR-4417 in the supernatant of PC3. These microRNAs have previously been identified as upregulated in CRPC tissue compared to hormone-sensitive PCa and benign prostatic hyperplasia (BPH). Proteomics-based analysis of biological processes in LNCaP and PC3 cells overexpressing these miRs demonstrated their involvement in tumor progression and cell proliferation^22^. In our study, we observed increased intracellular expression of both miR-3687 and miR-4417 in LNCaP cells cultured in a microphysiological system (MPS), which, although circumstantial, mirrors PCa progression. However, in terms of extracellular expression, only miR-3687 is downregulated in LNCaP cells. Considering that LNCaP cells are androgen-sensitive, the deregulation of both miR-3687 and miR-4417 in PC3 cells could indicate the presence of androgen-insensitive prostate cancer cells when both miRs are concurrently deregulated in circulation, whereas the deregulation of miR-3687 alone may indicate the hormone-sensitive phenotype. Although circumstantial, these findings further highlight the changes introduced by MPS in PCa and CRPC cell lines in vitro. Circulating microRNAs have long been considered a potential biomarker for prostate cancer. Not only as diagnostic tools but also to inform about disease progression, risk of CRPC onset, and monitoring the efficacy of treatments. This MPS model offers a novel tool to investigate the secretion of microRNAs in vitro and further unravel their pathophysiology.

With the characterization of this PCa-MPS model using conventional cell lines, we have shown the potential of using an advanced in vitro system to study phenotypical changes and the secretion of microRNAs in PCa and CRPC cells. MPS models, such as the TissUse HUMIMIC chip, are limited by low experimental throughput, however, when employed to address specific research questions can act as a powerful tool. The use of methodologies, such as next generation sequencing (NGS) to map the expression of non-coding RNA has been shown to substancially increment the experimental output of MPS-based experiments^43^. These approach can generate comprehensive data-sets from a limited number of samples, therefore maximizing the utility of complex and labor-intensive micro-fluidic platforms. Further studies using this system can investigate the secretion of microRNAs during and after the exposure of PCa and CRPC cells to approved therapies, such as abiraterone and enzalutamide. Alterations in microRNA secretion over time can be accessed during induced androgen resistance in PCa cells^44^. This model can also offer advantages in studying circulating tumor cell clusters (CTCs) as prognostic tools for prostate cancer (PCa)^45^. CTCs are linked to cancer metastasis, and the integration of PCa tissue explants derived from patient biopsies into a microphysiological system (MPS) holds potential for investigating the origins of CTCs and their correlation with PCa progression. Utilizing patient material in MPS provides an additional advantage as it preserves the heterogeneity of PCa and enables de facto ex vivo culture. By providing a more physiologically relevant model, PCa-MPS can potentially overcome the limitations of current in vitro models, nonetheless, comprehensive characterization efforts are warranted to understand the boundaries of hormone-sensitive and insensitive prostate cancer cells in dynamic cultures.

## Materials and methods

### Cell culture and microphysiological system preparation

Androgen-sensitive LNCaP cells and androgen-insensitive PC3 cells (DSMZ, Braunschweig, Germany) were used as PCa and CRPC models respectively. All cell culture supplements and media were acquired from PAN Biotech (Aidenbach, Germany) unless stated otherwise. Cells were cultured in RPMI media supplemented with 10% fetal bovine serum (FBS), 1% sodium pyruvate (100 mM), and 1% penicillin/streptomycin (10000U/mL). In conventional cultures (2D), cells were seeded in 24-well microplates at a density of 100 000 per well and kept in culture for 4 days. For 3D static culture, cells were initially embedded in hydrogels consisting of a mixture of 100µL 2% agarose, 75µL culture media containing 100 000 cells, and 25µL low-density rat-tail collagen I (Corning Life Sciences, Corning, NY, USA), in 1.5 mL tubes and polymerized at room temperature for 1.5-h. Subsequently, the gels were removed from the tubes and placed in 24-well microplates, with 500µL of culture media. The HUMIMIC chip2 MPS (TissUse, Berlin, Germany)^46,47^ was used to culture LNCaP and PC3 cells under dynamic conditions (Fig. 1). Individual gels were added to the culture chambers of the MPS chip, with 2 gels per perfusion circuit. 250µL of media was added to each culture chamber and chips were perfused at a frequency of 1 Hz for 4 days. Subsequently, supernatant and cell samples were collected for analysis from both conventional, 3D static, and dynamic MPS cultures.

### Immunofluorescence characterization

Cells grown in 2D or MPS were fixed with a 2% paraformaldehyde solution and subsequently permeabilized with a 0.1% Triton-X solution in Hank's Balanced Salt Solution (HBSS). Primary antibodies were incubated overnight at 4 °C in a solution of 0.1% Triton-X and 1% BSA (v/v) in HBSS. Secondary antibodies were incubated for two hours at room temperature the following day and samples were washed in the 0.1% Triton-X and 1% BSA (v/v) HBSS solution before imaging using a Keyence BZ-9000 fluorescent microscope (Keyence, Osaka, Japan). The antibody staining conditions used are described in Supplementary Information Table 1.

### Gene and microRNA expression

Total RNA was isolated from all culture conditions using Trizol-LS (TZ; ThermoFisher, Waltham, MA, USA), for gene and microRNA expression analysis. For 3D static and MPS conditions, 2 gels were combined to obtain one sample, in the MPS the gels shared the same perfusion circuit. Gels were lysed using 750 µL TZ per 250 µl sample volume, with 2% TritonX-100 added, and the lysate and homogenized by resuspending using a 20G needle, followed by a 20-min incubation at room temperature. 100% Ethanol (1 × lysate volume) was added to the TritonX-100/lysate mix and, subsequently, RNA and microRNA isolation were performed using the miRNeasy Mini Kit (Qiagen, Hilden, Germany), according to manufacturer specifications. For gene expression, RNA reverse transcription (RT) was performed using PrimeScript RT Master Mix (Takara, Kusatsu, Japan). The expression of microRNA was determined using stem-loop RT-qPCR analysis^8,22^. Details on the description of the stem-loop RT reaction are depicted in Supplementary Information Tables 4 and 5. The quantification of both gene and microRNA levels by PCR was subsequently performed using the SsoAdvanced™ Universal SYBR green (BioRad, Hercules, CA, USA) amplification protocol, according to manufacturer specifications. The housekeeping genes GAPDH and RNU6 were used as references for the gene and microRNA expression analysis, respectively. All primers used for PCR analysis were acquired from Microsynth (Balgach, Switzerland) and are listed in Supplementary information (Tables 2 and 6).

### PSA analysis

MPS supernatant samples were collected at different time points and stored at − 80 °C. PSA levels were determined by enzyme-linked immunosorbent assay (ELISA) using the Human Kallikrein 3/PSA DuoSet (R&D Systems, Minneapolis, MN, USA), according to the manufacturer's specifications.

### Data analysis

Gene and microRNA expression data were analyzed using GraphPad Prism 5.00 (GraphPad Software, La Jolla, CA, USA) to determine statistically significant differences between convention and MPS culture conditions. Experimental groups were compared using a two-tailed unpaired t-test with a confidence interval of 95%. The 2D culture samples were used as the reference group and the analysis was performed based on the ΔΔCt values^48^.

## Supplementary Information

Supplementary Information.

## Data Availability

The datasets generated for the current study are available upon request from the corresponding authors.
