# Supplementary material for: Microfluidic-based prostate cancer model for investigating the secretion of prostate-specific antigen and microRNAs in vitro
Source: Sci Rep. 2023 Jul 19;13:11623. doi: 10.1038/s41598-023-38834-y (PMC10356943; doi:10.1038/s41598-023-38834-y)
Supplement: Supplementary file 1 — Supplementary Information. [file 41598_2023_38834_MOESM1_ESM.pdf]

Supplementary information

Supplementary Table 1: Antibodies used for immunofluorescent characterization

Primary antibodies	Dilution		Supplier	Notes
	2D	MPS		
EGFR	1:1000	1:200	Abcam (#Ab30)	Membrane receptor
ZO1			BD biosciences (#610966)	Adhesion protein
**Secondary antibodies**				
Alexa Fluor 555 anti-mouse	1:500	1:200	Invitrogen (#A21424)	-
**Co-satins**				
Phalloidin488	1:1000		Abcam (#Ab176753)	Cytoskeleton stain
Hoechst33342			Merck (#14533)	Nuclear satin

Supplementary Table 2: gene expression primers

Gene	Forward	Reverse
GAPDH	5'-TCAAGGCTGAGAACGGGAAG-3'	5'-TGGACTCCACGACGTACTCA-3'
AR	5'-TGCCTGATCTGTGGAGAT-3'	5'-CGAAGACGACAAGATGGACA-3'
PSA	5'-CCGGAGAGCTGTGTCACCAT-3'	5'-GTGCAGCACCAATCCACGTC-3'
PSMA	5'-CCAAGTATTCCTGTTCATCCAATT G-3'	5'-CTTTGAGACTTCCTCTCCAGCTG-3'
CK5	5'-TCAAGACCCTCAACAATAAGTTTGC-3'	5'-TGCTCCTGCAGCAGGGTCCAC-3'
CK8	5'-CTGGAGGCGGAGCTTGGCAAC-3'	5'-AATTCGTTCTCCATCTCTGTACGC-3'
CK19	5'-AGATCGAAGGCCTGAAGGAAGAG-3'	5'-CCTCCACACTGACCTGGCCTC-3'
EpCam	5'-AGGTCCTCGCGTTCGGGCTTC-3'	5'-AGCAGTTTACGGCCAGCTTGTAG-3'
TPD52	5'-GAGGAAGGAGAAGATGTTGC-3'	5'-GCCGAATTCAAGACTTCTCC-3'
EGFR	5'-AGGCACGAGTAACAAGCTCAC-3'	5'-ATGAGGACATAACCAGCCACC-3'
ZO1	5'-CAACATACAGTGACGCTTCACA-3'	5'-CACTATTGACGTTTCCCCACTC-3'

Supplementary Table 3: RT thermocycler program for gene expression

Step	Temperature (C)	Duration (sec)	Description
#1	37	900 (15min)	Reverse-transcription
#2	85	5	Inactivation
#3	4	-	Cooling

Supplementary Table 4: Stem-loop RT reaction for microRNA expression

	stock concentration.	final concentration.	volume (µl)
RNA sample	100 ng/ µl	200 ng	2
SL-RT-miR-forward	1 µM	50 nM	0.75
SL-RT-miR-reverse	1 µM	50 nM	0.75
dNTP mix	2,5 mM	0,25 mM	1,5
RT-buffer	5x	1x	3
M-MLV-RTase	200U/µl	75 U (5U/µl)	0.375
RNase inhibitor	40 U/µl	3,75 U (0,25U/µl)	0.1
Milli Q water			6.525
Final volume / sample			*15*

Supplementary Table 5: Stem-loop RT thermocycler program

Step	Temperature (C)	Duration (min)	Description
#1	52	-	Lid
#2	16	30	
#3	42	30	Reverse-transcription
#4	85	5	Inactivation
#5	10	-	Cooling

Supplementary Table 6: microRNA primers

**Stem-loop RT primers**		
miR-3687	5'-CTCGTATCC AGTGCAGGGTCCGAGGTATTCGCACTGGATACGAGACGTCG-3'	
miR-4417	5'-GTCGTATCCAGTGCAGGGTCCGAGGTATTCGCACTGGATACGACCCCTCC-3'	
miR-26a	5'-GTCGTATCCAGTGCAGGGTCCGAGGTATTCGCACTGGATACGACAGCCTA-3'	
miR-205	5'-GTCGTATCCAGTGCAGGGTCCGAGGTATTCGCACTGGATACGACCAGACT-3'	
RNU6	5´-GTCATCCTTGCGCAGG-3´	
**qPCR primers**	**Forward**	**Reverse**
miR-3687	5'-TGCAAGCCCGGACAGGCGTTCGT-3´	5'-GTGCAGGGTCCGAGGT-3´
miR-4417	5'-TGCAAGGGTGGGCTTCCCGGAG-3´	
miR-26a	5'-GCCCGCTTCAAGTAATCCAGGAT-3´	5'-GTGCAGGGTCCGAGGT-3´
miR-205	5'-TGCCAGTCCTTCATTCCACCGG-3´	
RNU6	5'-CGCTTCGGCAGCACATATAC-3´	5'-AGGGGCCATGCTAATCTTCT-3´

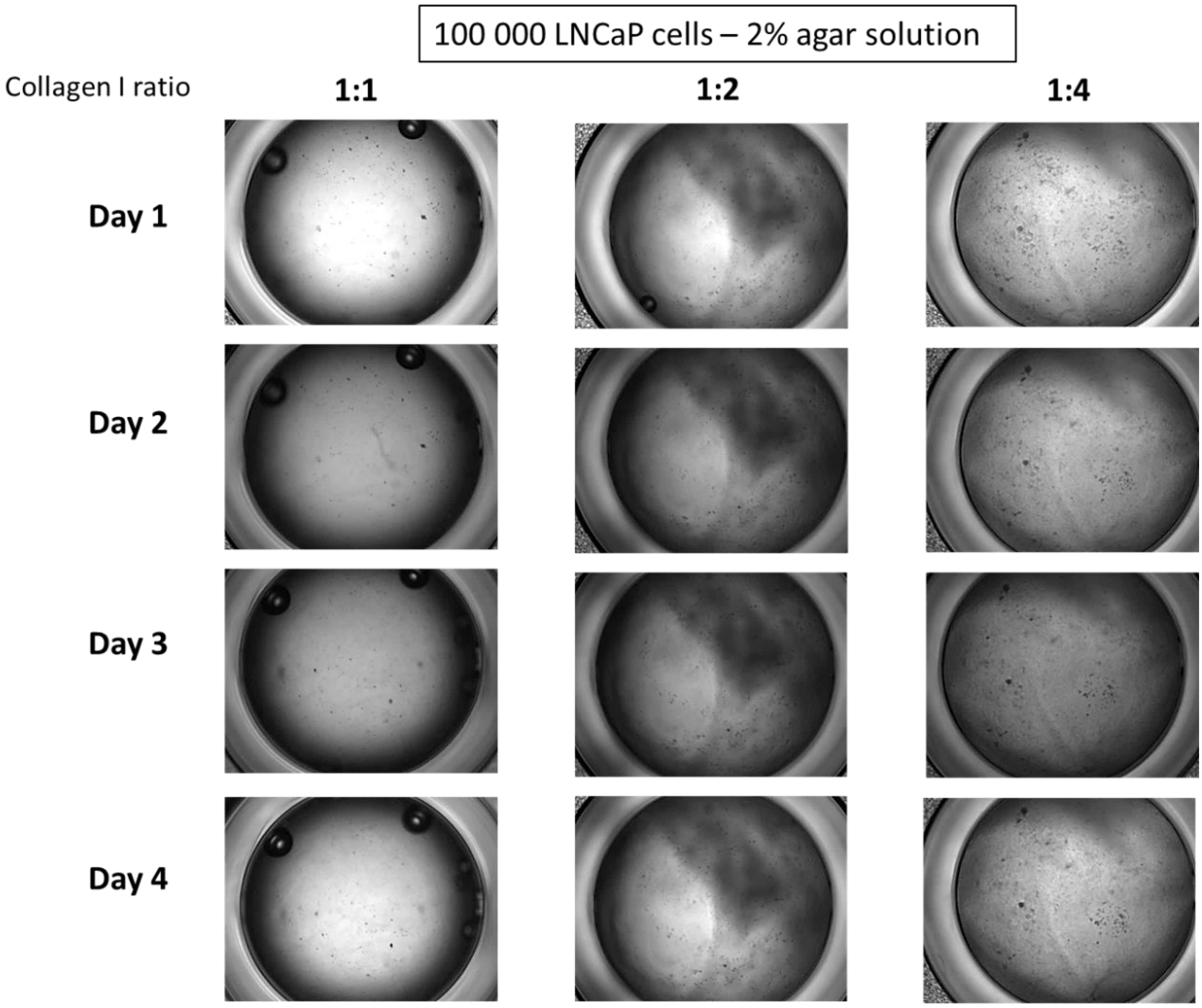

Supplementary Figure 1: Representative images of the agar/collagen hydrogel optimization. Different collagen rations in a 2% agar solution (v/v) in cell culture media, imbedded with 100 000 LNCaP cells. The ration 1:4 (75uL agar / 25uL collagen I) was determine to provide the most consistent cell growth and desirable structural integrity.
